# PGCNet: a Transformer–CNN hybrid segmentation model for pine wilt disease identification

**DOI:** 10.3389/fpls.2026.1760648

**Published:** 2026-01-28

**Authors:** Jiying Liu, Yaping Zhang, Xu Chen

**Affiliations:** 1 School of Information Science and Technology, Yunnan Normal University, Kunming, China; 2 Faculty of Geography, Yunnan Normal University, Kunming, China; 3 The Engineering Research Center of Geographic Information System Technology in Western China, Ministry of Education, Yunnan Normal University, Kunming, China

**Keywords:** feature fusion, pine wilt disease, plant disease detection, semantic segmentation, UAV remote sensing

## Abstract

Pine wilt disease, often referred to as the “cancer of pine trees,” is characterized by its rapid spread and extremely high mortality rate, posing a severe threat to forest ecosystems. Currently, most automatic identification methods for pine wilt disease based on UAV remote sensing imagery rely on a single architecture of Convolutional Neural Networks (CNNs) or Transformer, which suffer from limitations such as restricted receptive fields, insufficient global context modeling, and loss of local details. Existing fusion strategies typically adopt simple stacking or parallel designs without an effective hierarchical feature interaction mechanism, resulting in inadequate integration of semantic and detailed information, as well as high computational overhead, which hinders their deployment in edge computing environments. To address these issues, this study proposes PGCNet, a semantic segmentation model that efficiently fuses CNN and Transformer representations. The model employs CSWin Transformer as the backbone network to capture comprehensive global contextual information. A Progressive Guidance Fusion Module (PGFM) is designed to achieve effective cross-layer fusion of semantic and detailed features through a spatial–channel collaborative attention mechanism. Furthermore, a lightweight Context-Aware Residual Atrous Spatial Pyramid Pooling module (CAR-ASPP) is introduced to enhance multi-scale feature representation while significantly reducing the number of parameters and computational complexity. Experimental results demonstrate that PGCNet outperforms mainstream semantic segmentation models across multiple evaluation metrics, showing especially strong performance in scenarios with complex background interference and small-scale disease target identification. The proposed model achieves high accuracy with excellent computational efficiency, offering a practical solution for real-time monitoring and edge deployment of forestry disease detection, and exhibiting strong potential for extension to agricultural remote sensing disease identification tasks.

## 1 Introduction

As a keystone species in forest ecosystems, the health of pine trees directly influences biodiversity and ecological stability Yu et al. (2021b). Among the threats to pine forests, Pine Wilt Disease (PWD)—a devastating disease caused by the nematode Bursaphelenchus xylophilus—has emerged as one of the most destructive. The disease is primarily transmitted by the pine sawyer beetle (Monochamus alternatus) and characterized by its rapid spread, causing tree mortality within three months, and by its extremely high fatality rate Douda et al. (2015). If the removal of infected trees is delayed or detection efforts are insufficient, the epidemic can spread even more rapidly Yu et al. (2021b); Ichihara et al. (2000). Furthermore, global climate warming has reduced the stability of forest ecosystems, exacerbating both the speed and range of PWD transmission. Rising temperatures have facilitated the expansion of the disease into higher altitudes and latitudes Douda et al. (2015). Therefore, developing efficient detection technologies is of great significance for controlling the spread of PWD, protecting pine forest resources, mitigating economic losses, and maintaining ecological balance.

Currently, there are three primary methods for detecting trees infected by Pine Wilt Disease (PWD): field surveys, satellite remote sensing, and unmanned aerial vehicle (UAV) remote sensing Xu et al. (2023). Field surveys are time-consuming, inefficient, and prone to missing infected targets. Satellite remote sensing, on the other hand, is limited by its spatial resolution and temporal frequency, making it difficult to meet the requirements of high-precision detection. In recent years, UAVs equipped with high-resolution sensors have been widely employed in PWD monitoring. Owing to their flexibility, remote operability, and capability to capture images at appropriate altitudes, UAVs have become one of the most effective tools for monitoring PWD outbreaks Cai et al. (2023). Although UAV-based monitoring demonstrates great potential for PWD detection, efficiently and accurately identifying and extracting disease-related information from high-resolution UAV imagery remains a pressing challenge.

In terms of UAV image analysis, traditional machine learning algorithms have shown limited performance in disease detection tasks due to their reliance on handcrafted features. In contrast, deep learning (DL) has gradually become the mainstream approach, owing to its outstanding performance in image segmentation and object detection tasks Ding et al. (2025). For instance, Run Yu et al. utilized high-resolution UAV imagery of pine wilt disease and successfully identified various infection stages by combining Faster R-CNN and YOLOv4 networks Yu et al. (2021b). Similarly, Xinquan Ye et al. employed an improved YOLOv5 in conjunction with the StrongSORT algorithm to achieve visual tracking and counting of infected trees in complex forest environments, providing new insights for intelligent disease monitoring and management Ye et al. (2024). However, bounding box–based object detection models (e.g., YOLO) struggle to delineate the precise boundaries of infected regions and are incapable of quantifying infection severity Wu and Jiang (2023); Xia et al. (2021). In contrast, semantic segmentation models, which perform pixel-level classification, can achieve accurate extraction and grading of disease-affected areas Wu and Jiang (2023); Xia et al. (2021). Consequently, the methodological shift from “detection” to “segmentation” has become a key direction for enhancing the fine-grained monitoring capability of Pine Wilt Disease (PWD).

Among deep learning–based semantic segmentation algorithms, Convolutional Neural Networks (CNNs) have become the dominant technology for Pine Wilt Disease (PWD) identification due to their outstanding performance in image recognition tasks Wu and Jiang (2023). CNNs can automatically extract key features such as texture, color, and morphology through convolutional operations, thereby avoiding the labor-intensive process of handcrafted feature construction. For example, Yongjun Ding et al. employed an improved DeepLabV3+ network to segment apple leaf lesions, effectively addressing critical challenges such as blurred lesion boundaries, low pixel proportions, and highly variable lesion morphology Ding et al. (2025). Zhenyu Wu et al. proposed a detection and extraction algorithm based on Mask R-CNN, achieving effective delineation of PWD-infected regions Wu and Jiang (2023). However, as the depth of CNNs increases, their computational cost grows significantly. Moreover, CNNs primarily focus on local feature extraction, leading to insufficient global modeling capability when dealing with diseases like PWD, which exhibit both local and global diffusion characteristics and often occur against complex background conditions Wu et al. (2025).

Owing to its ability to capture long-range dependencies, the Transformer architecture has been widely adopted in semantic segmentation, becoming a pivotal trend in computer vision. The Vision Transformer (ViT) Dosovitskiy (2021), which divides an image into sequences and models pixel-wise dependencies through the attention mechanism, can effectively capture global contextual information. Huan Liu et al. proposed a novel Cluster Transformer and D-Cluster Transformer, which achieved effective identification of Pine Wilt Disease (PWD) Liu et al. (2024a). However, Transformers also have inherent limitations, including the need for large-scale training datasets, high computational costs, and insufficient ability to process local details Chen et al. (2025). Although Transformer-based networks can effectively capture global dependencies and often produce results comparable to or even superior to those of CNNs, they tend to degrade local feature representation, leading to the loss of fine-grained spatial details Cui et al. (2025).

To address the aforementioned limitations of CNNs and Transformers, researchers have attempted to combine the strengths of both architectures by constructing hybrid networks for semantic segmentation tasks, achieving remarkable progress in applications such as crop and forest disease detection. Qiangjia Wu et al. enhanced the segmentation accuracy of Pine Wilt Disease (PWD) by alternately stacking CNN and Transformer modules within the backbone network and introducing additional Transformer blocks into the encoder Wu et al. (2025). However, existing approaches that integrate CNNs and Transformers are often based on simple stacking or parallel fusion, lacking hierarchical feature interaction mechanisms. As a result, shallow detail features and deep semantic features are not fully fused. Moreover, such complex fusion structures usually entail high computational overhead, which hinders their deployment on edge devices.

In response to the aforementioned challenges, this study proposes PGCNet (Progressive Guidance and Context Network), a hybrid semantic segmentation model that integrates CNN and Transformer architectures. Specifically, CSWin Transformer Dong et al. (2022) is adopted as the backbone feature extraction network to capture comprehensive global image representations, while CNN components serve as auxiliary modules to compensate for the loss of local details. The resulting multi-scale features are then effectively fused to achieve robust and accurate disease segmentation. The proposed model achieves fast and precise detection of Pine Wilt Disease (PWD) while maintaining high computational efficiency, providing a practical solution for deployment on forest edge devices.

The main contributions of this paper are summarized as follows:

- To address the challenges of large target scale variation and complex backgrounds in UAV-based PWD imagery, CSWin Transformer is introduced as the backbone network, providing rich and diverse multi-scale representations for subsequent feature enhancement and fusion.
- To resolve the gap between deep features (rich in semantics but lacking fine details) and shallow features (rich in details but semantically weak), we propose a top-down hierarchical fusion architecture (PGFM). By incorporating a Spatial–Channel Synergistic Attention (SCSA) mechanism, PGFM progressively guides multi-level feature interaction and fusion, resulting in high-resolution feature maps with both strong semantics and rich structural details. This design effectively suppresses background noise and significantly improves segmentation accuracy in infected regions.
- A Context-Aware Residual Atrous Spatial Pyramid Pooling (CAR-ASPP) module is developed to reduce computational overhead while retaining multi-level semantic information through a residual structure. This module efficiently models and aggregates contextual information at different scales and is applied only to the final output features. On the one hand, the last-layer features contain the most concentrated semantic information, making them suitable for multi-scale context modeling; on the other hand, this design avoids the redundant computational cost that would result from inserting the module at multiple network stages.

## 2 Materials and methods

### 2.1 Study area and data acquisition

**Figure 1:**
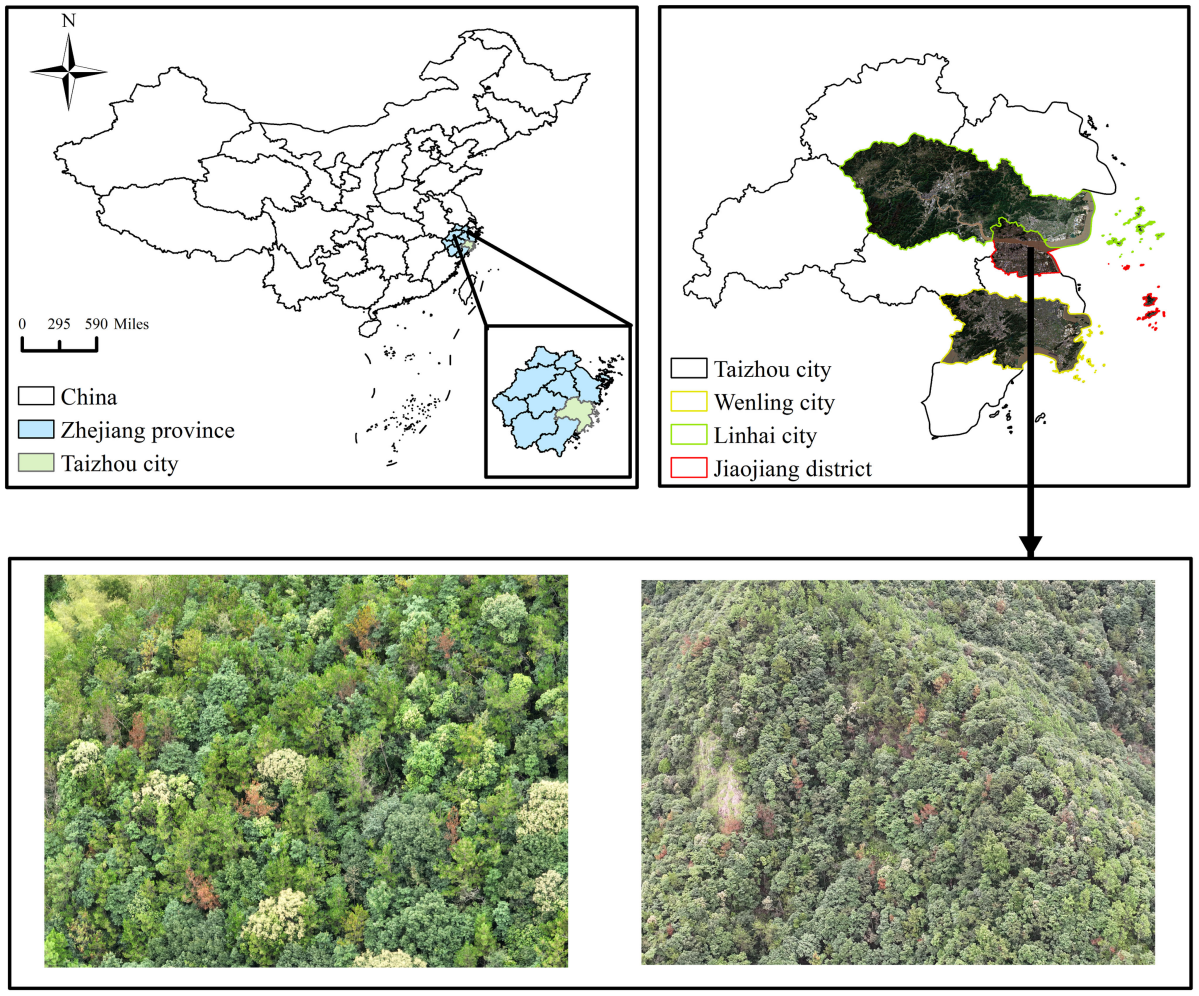
Map of the study area.

The dataset used in this study was collected from Taizhou City, Zhejiang Province, China $\left(28^{\circ }01^{\prime }–29^{\circ }20^{\prime }\text{ N},120^{\circ }17^{\prime }–121^{\circ }56^{\prime }\text{ E}\right)$. Taizhou is characterized by a subtropical monsoon climate with distinct seasonal variations. The average temperature in winter remains above 0°C, while the average summer temperature exceeds 20°C, and the annual precipitation is greater than 1000 mm. The forest coverage rate in Taizhou is approximately 60.3%, with a total forested area of about 6.9 million mu (≈ 4600 km^2^). The dominant tree species include Masson pine (Pinus massoniana), Chinese fir (Cunninghamia lanceolata), and broad-leaved forests. Pine trees are widely distributed across the mountainous areas of Taizhou. In this study, UAV image data were mainly collected from Jiaojiang District, Wenling City, and Linhai City, where field surveys confirmed the presence of pine trees at varying stages of Pine Wilt Disease (PWD) infection. The distribution of the study areas is illustrated in **Figure 1**.

### 2.2 Data acquisition and preprocessing

#### 2.2.1 UAV image acquisition

In this study, aerial imagery of Pine Wilt Disease (PWD)-affected forest areas was captured using a DJI UAV, DJI Phantom 3 (DJI, China). To establish a multi-scale observation system, flight routes were planned with multi-angle trajectories, and the flight altitude was set between 50m and 100m. Higher-altitude flights (80–100m) provided broad forest canopy overviews, whereas lower-altitude flights (50–70m) enabled high-resolution imaging of individual infected pine trees scattered within the forest. To ensure data diversity and comprehensiveness, UAV missions were conducted under varied environmental conditions, including different land cover types such as bare soil, rocky areas, standing deadwood zones, and broad-leaved forest belts, as well as under sunny, cloudy, and overcast weather conditions. In total, 376 original high-resolution UAV images were collected, each with a spatial resolution of 5000 × 4000 pixels.

#### 2.2.2 UAV image preprocessing

Initially, the collected UAV images were screened to remove samples without Pine Wilt Disease (PWD), resulting in a final set of 90 high-resolution images. Considering the progression of Pine Wilt Disease (PWD), visual symptoms manifest as a distinct transition in needle coloration. In this study, based on the capabilities of RGB imagery, the visible infection process is specifically classified into the early stage (initial discoloration to yellowish-brown or dull green) and the mid-to-late stage (complete reddish-brown or grayish-white discoloration). Note that the asymptomatic stage is excluded from this study, as identifying latent infection typically requires hyperspectral sensors. Based on these visual criteria, infected regions across both stages were precisely annotated using LabelMe software. Given that high-resolution images can impose significant computational overhead during model training, and to both enhance model adaptability and augment the dataset, the images were cropped using a sliding window approach. The window overlap was set to 10 pixels, and any resulting blank images were discarded. After preprocessing, a total of 1,268 images with a size of 512 × 512 pixels were obtained.

### 2.3 Dataset construction

To further increase the dataset size and improve the generalization ability of the model, data augmentation was applied to the existing dataset. To prevent data leakage, the original dataset was first split into training and validation sets at an 8:2 ratio. Data augmentation was then applied independently to each subset.

To avoid overfitting due to excessive duplication of augmented data, a combination of multiple augmentation strategies was employed:

1. Geometric transformations: Each image was randomly subjected to either a horizontal flip or a vertical flip.
2. Photometric adjustments: With an 80% probability, the image brightness and contrast were adjusted to simulate varying lighting conditions.
3. Noise injection: With a 40% probability, Gaussian noise was added to the images to enhance the model’s robustness to image complexity.

**Figure 2:**
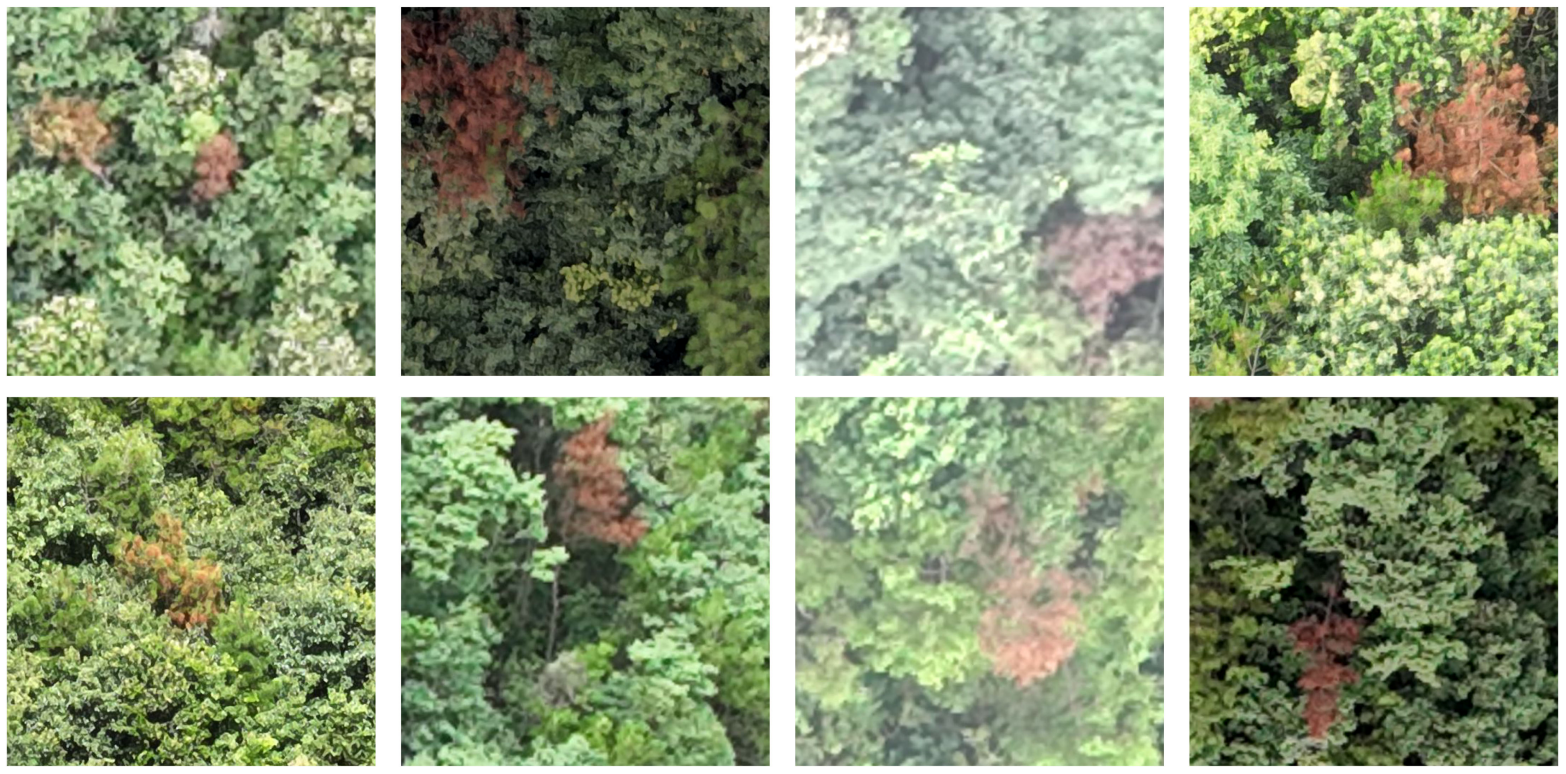
Examples from the PWD dataset.

After augmentation, the training set contained 2,050 images, and the validation set contained 509 images, as illustrated in **Figure 2**.

### 2.4 Methods

#### 2.4.1 Overall framework of the PGCNet

**Figure 3:**
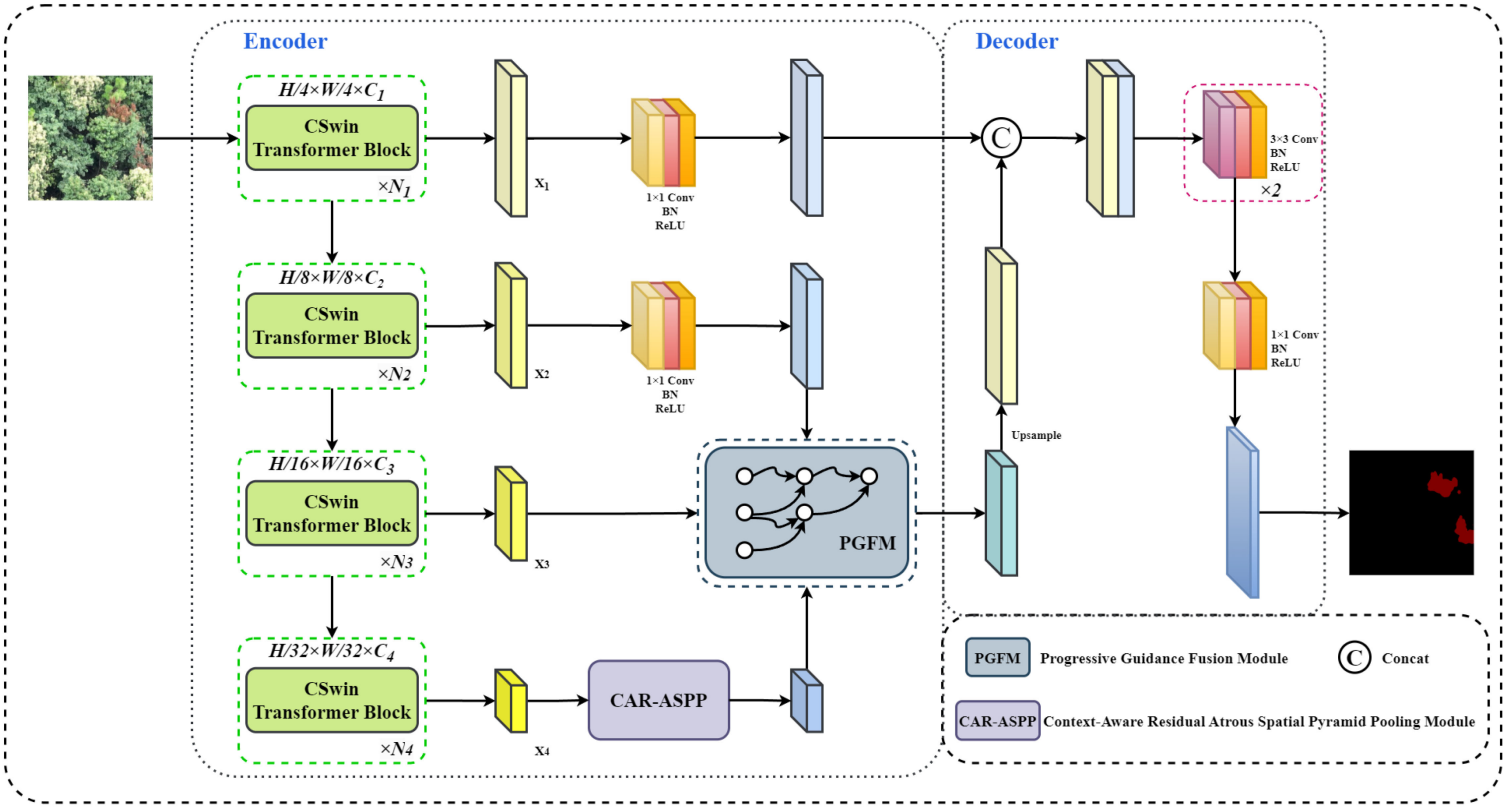
Overall architecture of the PGCNet model.

In the task of Pine Wilt Disease (PWD) identification, infected pine trees exhibit diverse visual features depending on the degree of disease progression. Coupled with the multi-scale characteristics of high-resolution UAV imagery, this poses significant challenges for traditional segmentation methods to achieve accurate recognition. To address this technical challenge, we developed PGCNet (Progressive Guidance and Context Network), a semantic segmentation model for PWD. PGCNet adopts a CSWin Transformer–CNN hybrid encoder-decoder architecture, enabling precise and efficient segmentation under complex background conditions. The overall architecture of the network is illustrated in **Figure 3**.

In the encoder, CSWin Transformer is employed as the backbone to capture global contextual features of infected pine trees, leveraging its capability to model long-range dependencies. Additionally, a Context-Aware Residual Atrous Spatial Pyramid Pooling (CAR-ASPP) module is introduced, which combines multi-scale depthwise separable atrous convolutions with a channel attention mechanism to expand the receptive field, reduce computational overhead, and fuse global and local contextual information. Residual connections further enhance feature representation and mitigate information loss. Moreover, a Progressive Guidance Fusion Module (PGFM) is designed to gradually integrate deep semantic features with shallow detail features, preserving fine boundary information while enhancing the semantic discriminability of targets. This design effectively suppresses background interference and improves the model’s ability to accurately recognize PWD-affected trees.

In the decoder, inspired by the DeepLabV3+ Chen et al. (2018) decoder structure, two consecutive 3×3 convolutions are first used to integrate and extract the fused features, followed by a 1×1 convolution to adjust the feature dimension. Finally, bilinear interpolation is employed to upsample the output feature maps to the original image resolution. This architecture significantly enhances multi-scale feature representation and semantic-detail preservation for PWD, while maintaining parameter efficiency, providing a novel technical solution for precise UAV-based forest disease monitoring.

#### 2.4.2 Backbone feature extraction network: CSWin transformer

When processing UAV-acquired images of Pine Wilt Disease (PWD) in pine trees, disease features are typically irregularly distributed and sparsely located across different regions of the canopy. Methods that rely solely on local modeling often fail to establish a unified recognition standard and cannot capture global contextual information, which limits segmentation accuracy. Compared with conventional Transformers (e.g., ViT) and Swin Transformer, CSWin Transformer introduces a Cross-Shaped Window Self-Attention mechanism that achieves a global receptive field while maintaining linear computational complexity. This architecture is particularly inherently suited for PWD detection. Unlike general natural images where objects are often centered and continuous, PWD-infected trees are typically sparsely distributed and exhibit irregular canopy shapes across the forest. Standard square-window attention limits the contextual modeling to local regions. In contrast, the cross-shaped window in CSWin effectively captures the scattered global context of the pine forest, ensuring that even discrete, small-scale infection targets are modeled within a broader environmental context. Therefore, in this study, CSWin Transformer Dong et al. (2022) is adopted as the backbone feature extraction network to enhance the model’s capability in modeling global semantic relationships, thereby improving the segmentation performance of diseased pine regions while maintaining computational efficiency.

**Figure 4:**
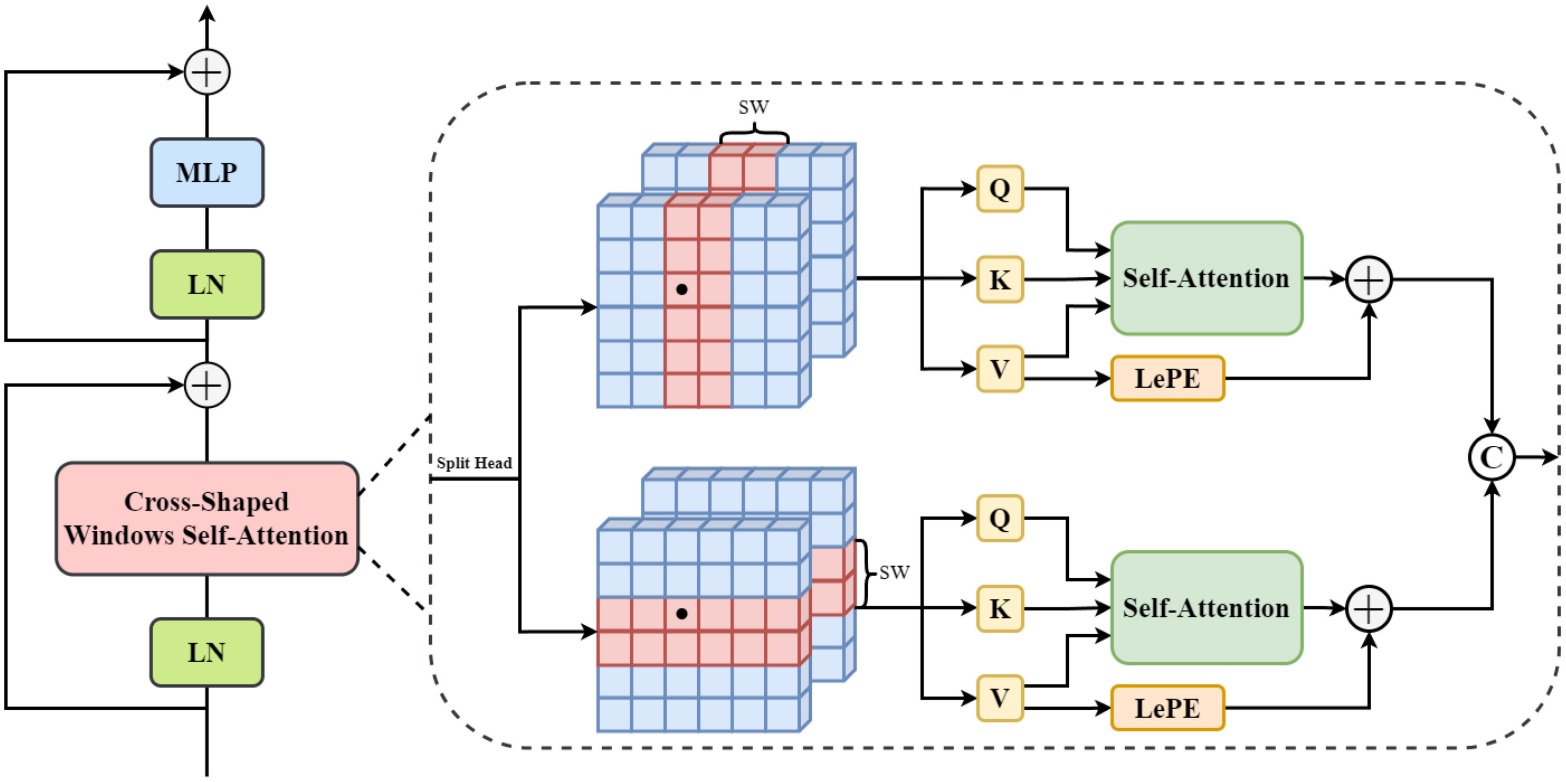
Internal structure of the CSWin transformer.

**Figure 4** illustrates the overall architecture of the CSWin Transformer. Its core module, the cross-shaped window self-attention, performs self-attention in horizontal and vertical stripes, forming a cross-shaped attention window. For an input feature map *X* ∈ *R^H^*^×^*^W^*^×^*^C^*, it is first projected linearly into queries (*Q*), keys (*K*), and values (*V*) for *K* attention heads, defined in Equation 1 as:

(1) $$\begin{array}{l} Q=XW^{Q},\;K=XW^{K},\;V=XW^{V} \end{array}$$

Where $W^{Q},W^{K},W^{V}\in R^{C\times d_{k}}$ are the projection matrices for queries, keys, and values, typically with dimensions $d_{k}=C/K$. The attention heads in CSWin are divided into two groups, performing self-attention in parallel along horizontal and vertical stripes. In the horizontal stripe, the feature map is divided into *M* non-overlapping horizontal bands. For the *K*-th attention head, the self-attention on the *i*-th band is computed in Equations 2 and 3 as:

(2) $$\begin{array}{l} Y_{i}^{k}=Attention(X_{i}W_{k}^{Q},X_{i}W_{k}^{K},X_{i}W_{k}^{V}) \end{array}$$

(3) $$\begin{array}{l} H\_Attention_{k}(X)= \end{array}[Y_{1}^{k},Y_{2}^{k},\ldots ,Y_{M}^{k}]$$

Similarly, the self-attention operation along the vertical direction is defined in Equation 4 as:

(4) $$\begin{array}{l} V\_Attention_{k}(X)= \end{array}[Z_{1}^{k},Z_{2}^{k},\ldots ,Z_{N}^{k}]$$

Finally, the outputs of the two heads are concatenated along the channel dimension and linearly projected by $W^{O}\in R^{C\times C}$ to integrate horizontal and vertical spatial information, yielding a unified representation with global contextual awareness. The formulation is given in Equation 5 as follows:

(5) $$\begin{array}{l} CSWin\_Attention(X)\\ =Concat(H\_Attention(X),V\_Attention(X))W^{O} \end{array}$$

This process fuses information from both horizontal and vertical spatial directions, enabling the model to build a unified global contextual representation. By partitioning the feature map along two directions to form cross-shaped windows, CSWin Transformer significantly enlarges the receptive field of each token, effectively establishing long-range dependencies. This property is particularly important for the recognition and fine-grained segmentation of diseased pine regions, enhancing model performance in scenarios where disease distributions are irregular and information is sparse.

#### 2.4.3 Context-Aware Residual Atrous Spatial Pyramid Pooling

**Figure 5:**
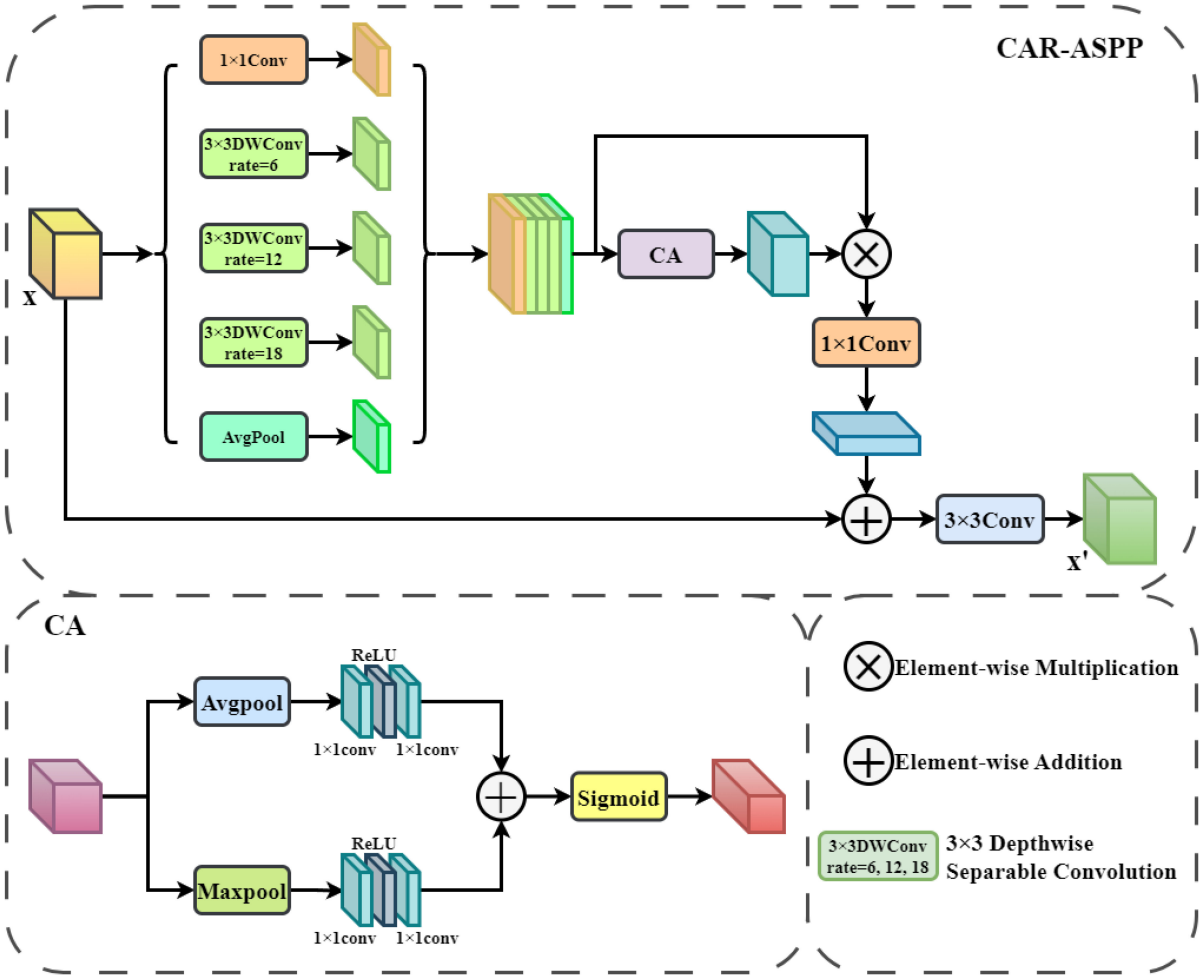
Architecture of the CAR-ASPP module.

Although the CSWin Transformer backbone provides excellent global feature modeling capability, Pine Wilt Disease (PWD) in UAV imagery exhibits significant multi-scale characteristics: small diseased patches occupying only a few pixels, as well as larger infection areas distributed across the canopy. This scale variation poses challenges for accurate detection. The Atrous Spatial Pyramid Pooling (ASPP) Chen et al. (2018), as a core module of DeepLabV3+, employs parallel multi-scale atrous convolutions, which helps adapt to the scale variations of PWD targets from UAV perspectives. However, the parallel processing of multi-scale atrous convolutions typically introduces additional computational overhead and inference latency, making it difficult to meet the real-time and lightweight requirements for UAV-based applications and edge deployment. To address this issue, we designed the Context-Aware Residual ASPP (CAR-ASPP) module. As shown in **Figure 5**, this module employs depthwise separable atrous convolutions, which expand the receptive field while significantly reducing computational cost.

Considering that UAV-acquired pine images often contain complex backgrounds and varying illumination, making it difficult to capture critical feature information, we further incorporate a channel attention mechanism combining global average pooling and max pooling after multi-scale feature fusion. This mechanism allows the network to aggregate both global semantic information and local details, thereby enhancing context-awareness. Residual connections are also employed to preserve input feature details, stabilize gradient propagation, and mitigate feature degradation and information loss during multi-scale semantic fusion. Through the combination of residual-based gradient optimization and channel-attention driven contextual awareness, the CAR-ASPP module effectively reduces computational overhead while ensuring robust extraction and representation of PWD features at multiple scales.

The multi-scale feature extraction structure of CAR-ASPP mainly consists of five branches and a fusion module. Let the input feature map be $x\in R^{C_{in}\times H\times W}$. First, the 1×1 convolution branch is employed to extract local features, as expressed in Equation 6:

(6) $$f_{1}=ReLU(BN(Conv_{1\times 1}(x)))$$

Here, ReLU(·) denotes the activation function, and BN(·) represents the feature normalization operation. Next, in the multi-scale depthwise separable dilated convolution branch, for each dilation rate $r_{i}\in \{r_{2},r_{3},r_{4}\},$ the following operation is defined in Equation 7:

(7) $$f_{i}=ReLU(BN(DWConv_{3\times 3}^{r_{i}}(x))),i=2,\;3,\;4$$

Here, $DWConv^{r}(\cdot )$ denotes a depthwise separable convolution with a dilation rate of *r*. The global average pooling branch is used to capture global contextual information, which is computed in Equation 8 as follows:

(8) $$f_{5}=ReLU(BN(Conv_{1\times 1}(GAP(x)))),f_{5}\in R^{C_{out\times 1\times 1}}$$

Here, *GAP*(·) denotes global average pooling. Additionally, the feature map obtained from global average pooling is resized via bilinear interpolation to facilitate fusion with other features.

After fusing the above output features, the channel attention (CA) mechanism Woo et al. (2018) is applied to enhance the model’s focus on target-related features. Finally, a 3×3 convolution is used to integrate the original features with the, as formulated in Equations 9 and 10:

(9) $$F=Concat(f_{1},f_{2},f_{3},f_{4},f_{5})$$

(10) $$x^{\prime }=Conv_{3\times 3}(Conv_{1\times 1}(CA(F)⊗F)⊕x)$$

Here, CA(·) denotes the channel attention mechanism, which applies both average pooling and max pooling on the input features. The pooled features are then passed through a convolution layer followed by a ReLU activation, and the resulting features are combined via element-wise addition. Finally, a Sigmoid activation is applied. The symbols ⊙ and ⊕ denote element-wise multiplication and addition, respectively.

#### 2.4.4 Progressive Guidance Fusion Module

To address the semantic-detail discrepancies present across different feature hierarchies in semantic segmentation, we propose the Progressive Guidance Fusion Module (PGFM). Deep features possess strong global semantic understanding, capturing the overall structure and category information of PWD targets; however, their low resolution limits the expression of fine details. Conversely, shallow features retain rich local textures and edge information, but lack global semantic discriminability. PGFM extracts shallow, intermediate, and deep features from the backbone network and performs hierarchical fusion in a progressive guidance manner. This allows deep semantic information to be gradually integrated into intermediate and shallow features, resulting in high-resolution feature maps that combine strong semantic representation with rich spatial details. To further enhance feature discriminability and suppress background responses, PGFM incorporates a Spatial-Channel Synergistic Attention (SCSA) module Si et al. (2025) after fusion. This module re-weights the fused features based on salient information, thereby improving the model’s segmentation robustness in complex backgrounds and its boundary representation capability.

**Figure 6:**
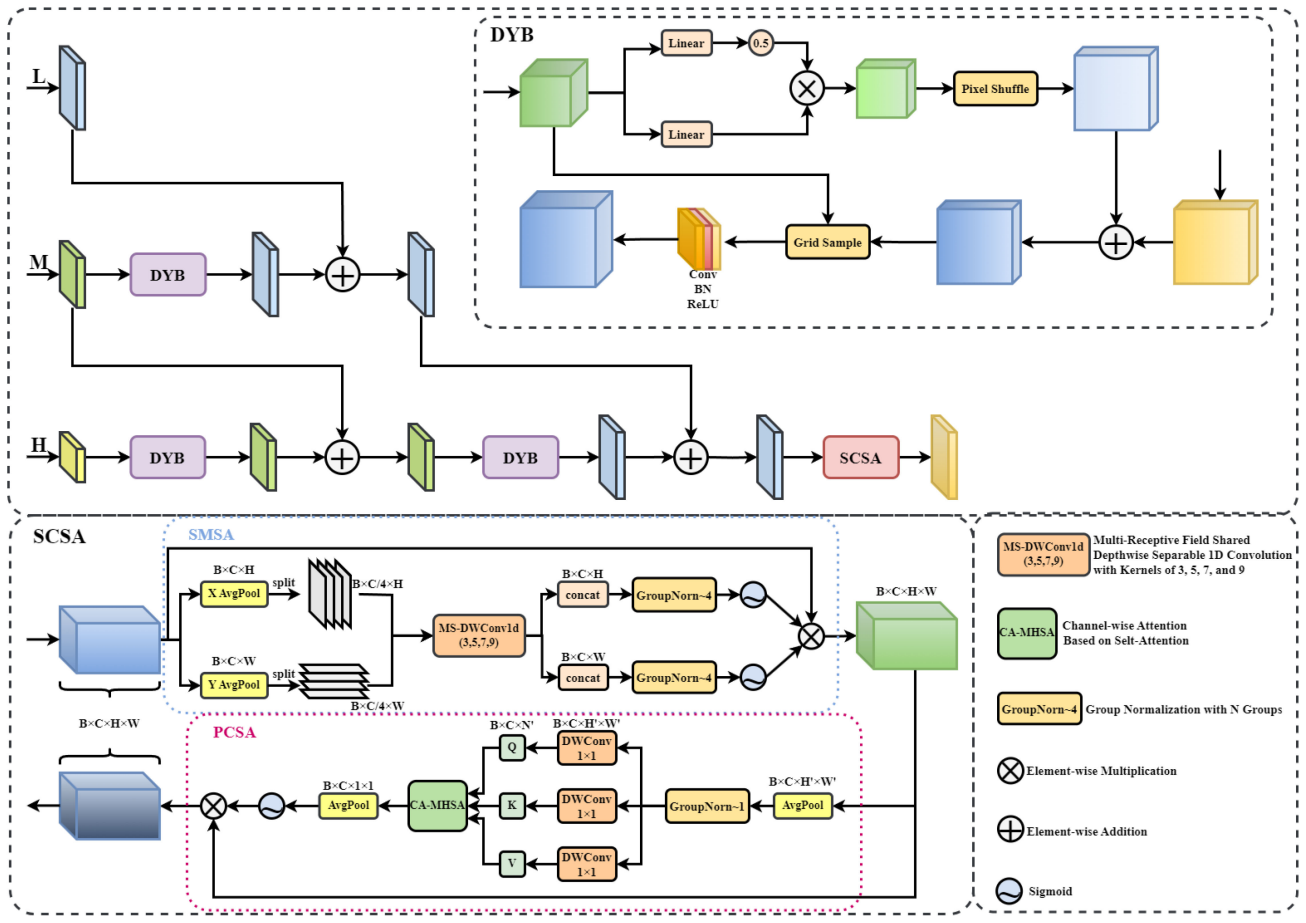
Architecture of the PGFM module.

As shown in **Figure 6**, the proposed PGFM adopts a top-down hierarchical fusion framework. It hierarchically fuses the deep-level features $H\in R^{C_{1}\times H_{1}\times W_{1}}$ extracted by the backbone network, the middle-level features $M\in R^{C_{2}\times H_{2}\times W_{2}},$ and the shallow-level features $L\in R^{C_{3}\times H_{3}\times W_{3}}$. The fusion computation process is described as is described in Equations 11–14 as follows:

(11) $$M_{0}=DY\;B(H)+M$$

(12) $$L_{0}=DY\;B(M)+L$$

(13) $$F=DY\;B(M_{0})+L_{0}$$

(14) f=SCSA(F)

Here, *DY B*(·) represents the feature map obtained after convolutional refinement following DySample Liu et al. (2023c) upsampling. PGFM uses *DY B*(·) to perform upsampling of features at different hierarchical levels. Unlike dynamic convolution-based upsampling methods such as CARAFE Wang et al. (2019) and SAPA Lu et al. (2022), DySample adopts a point sampling-centric approach, adaptively generating upsampling coordinates based on the content of the input feature map. As illustrated in the DYB module in **Figure 6**, the feature map is first transformed through a linear layer and multiplied by an offset factor to calculate the relative displacement coordinates for each pixel. Then, through a pixel shuffle operation, the feature map with displacement information is resized to the target upsampling size. The computed displacement coordinates are added to the base grid coordinates to determine the precise sampling location for each pixel, followed by grid-based sampling. This content-adaptive sampling strategy is critical for pine canopy segmentation. A major challenge in PWD identification is preserving the fine-grained needle-like textures that are easily blurred by traditional interpolation. DySample dynamically generates sampling points based on feature content, allowing the model to adaptively focus on the high-frequency structural details of pine needles. This significantly enhances the accuracy of boundary restoration and expansion, promoting more balanced feature fusion.

After fusing features from different hierarchical levels, the PGFM module applies a Spatial-Channel Synergistic Attention (SCSA) mechanism Si et al. (2025) to enhance the expressiveness of the fused features. As shown in **Figure 6**, SCSA sequentially combines Shared Multi-Semantic Spatial Attention (SMSA) and Progressive Channel Self-Attention (PCSA). SMSA employs multi-scale depthwise shared 1D convolutions to capture multi-semantic spatial information from the fused features, effectively integrating global contextual dependencies with multi-semantic spatial priors. PCSA computes channel similarity and contribution using compressed spatial knowledge, mitigating semantic discrepancies across spatial structures. This synergistic design addresses the high similarity between early-stage infected pines and background vegetation. Standard attention often fails to distinguish these subtle differences. By integrating spatial cues with channel information, SCSA effectively suppresses the response of irrelevant green background noise while highlighting the salient discoloration features of the target. By synergistically leveraging multi-semantic spatial and channel information, SCSA integrates spatial cues from different semantic hierarchies, enabling the model to simultaneously acquire global semantics and local details, thereby enhancing the feature representation capability of the network.

## 3 Experimental results and analysis

### 3.1 Implementation details

All experiments were conducted on a server equipped with an NVIDIA GeForce RTX 3090 GPU, running Ubuntu 18.04. The deep learning framework used was PyTorch 1.12.1 with CUDA 11.3, and the programming language was Python 3.9. The proposed PGCNet model, as well as all baseline comparison models, were trained and evaluated under the same hardware and software environment to ensure fair comparison.

For optimization, the Adam optimizer was employed for parameter updates. The training process used a batch size of 4, and the maximum number of epochs was set to 50, which was sufficient to achieve convergence. The initial learning rate was set to 0.0005. To improve training stability and performance, a Cosine Annealing learning rate schedule was applied, wherein the learning rate smoothly decreases following a cosine function as the number of training epochs increases, effectively mitigating gradient oscillations in the later stages of training.

During data preprocessing, all images were standardized. To enhance the generalization ability of the model, data augmentation techniques—including random cropping, horizontal flipping, and color jittering—were applied during training.

### 3.2 Evaluation metrics

This study aims to perform the segmentation of pine trees infected with PWD. Given that the infected regions (foreground) are typically much smaller in area than healthy trees or the background, evaluation based on the overall segmentation performance may be biased toward the background and fail to reflect the model’s true capability in identifying the foreground. Therefore, all evaluation metrics were calculated exclusively over the PWD-infected regions. The proposed model was quantitatively evaluated using the Intersection over Union (IoU), Precision, Recall, and F1-Score, defined in Equations 15–18 as follows:

(15) $$IoU=\frac{TP}{TP+FP+FN}$$

(16) $$Recall=\frac{TP}{TP+FN}$$

(17) $$Precision=\frac{TP}{TP+FP}$$

(18) $$F1=\frac{2\times Precision\times Recall}{Precision+Recall}$$

Here, True Positives (*TP*) represent the number of pixels correctly predicted as PWD-infected, False Positives (*FP*) denote pixels incorrectly predicted as infected when they are actually healthy or background, and False Negatives (*FN*) refer to pixels of infected trees incorrectly predicted as non-infected.

Intersection over Union (*IoU*) measures the ratio of the intersection to the union between the predicted and ground truth regions. Its value ranges from 0 to 1, with higher values indicating better overlap between the prediction and the ground truth.

*Precision* indicates the proportion of pixels correctly predicted as PWD-infected among all pixels predicted as infected by the model.

*Recall* represents the ratio of correctly predicted PWD-infected pixels to the total number of true infected pixels.

*F*1 − *Score* provides a harmonic mean of Precision and Recall, reflecting a balance between the two metrics.

### 3.3 Comparative experiments

In this study, PGCNet was compared with several mainstream semantic segmentation models on the constructed dataset, including UNet Ronneberger et al. (2015), PSPNet Zhao et al. (2017), HRNet Yu et al. (2021a), PSANet Zhao et al. (2018), SWin Transformer Liu et al. (2021), SegFormer Xie et al. (2021), Twins Chu et al. (2021), ConvNeXt Liu et al. (2022b), and Clusterformer Liu et al. (2024a). To ensure experimental fairness, all models were trained and evaluated under the same environment and parameter settings. All models converged after 50 epochs. The experimental results are analyzed from both quantitative and qualitative perspectives.

1) Quantitative Evaluation: **Table 1** presents the comparative results of the above models, where bold and underlined values indicate the best and second-best results, respectively.

**Table 1:** Comparison of different semantic segmentation models.

Model	Encoder	IoU (%) ↑	Recall (%) ↑	Precision (%) ↑	F1 (%) ↑	Params (M) ↓	FLOPs (G) ↓
DeepLabv3+ (CVPR2018)	Xception	77.94	84.68	**90.72**	87.60	54.71	243.31
UNet (MICCAI2015)	–	73.11	83.16	85.82	84.47	31.04	218.97
PSPNet (CVPR2017)	ResNet50	77.22	85.29	89.09	87.15	49.07	194.40
HRNet (CVPR2019)	HRNetv2_w18	78.17	87.60	87.90	87.75	9.64	18.66
Swin (ICCV2021)	Swin-T	70.44	79.08	86.57	82.66	59.83	234.90
PsaNet (ECCV2018)	ResNet50	77.28	85.56	88.87	87.18	59.13	200.07
Segformer (NeurIPS2021)	mixViT	76.27	84.20	89.01	86.54	**3.72**	**6.38**
Twins (ICCV2021)	PCPVT	71.95	81.26	86.27	83.69	27.84	37.00
ConvNext (CVPR2022)	ConvNext	79.33	86.72	90.31	88.47	60.13	233.50
Clusterformer (TGRS2024)	–	76.29	83.99	89.27	86.55	17.70	11.34
PGCNet (Ours)	CSWin	**79.75**	**88.50**	88.97	**88.73**	25.67	48.59

The bold values indicate the best results, and the underlined values represent the second-best results.

From the results, the proposed PGCNet demonstrates superior performance in IoU, Recall, and F1 metrics. Specifically, it achieves an IoU of 79.75%, which is 9.31% higher than the lowest-performing SWin Transformer. The Recall reaches 88.50%, the highest among all models, indicating a significant advantage in capturing the completeness of PWD-infected regions.

For Precision, PGCNet is slightly lower compared to DeepLabv3+, PSPNet, SegFormer, ConvNeXt and Clusterformer. This is due to the high similarity in texture and color features between the foreground (infected trees) and background in the study scenes, which may lead to over-prediction along boundaries, reducing Precision. However, this strategy significantly enhances Recall. Prioritizing Recall is particularly advantageous in disease detection scenarios, as it effectively mitigates potential risks caused by missed detections. Despite a relatively lower Precision, the high Recall and IoU result in the highest F1-score, demonstrating the model’s superior balance between overall segmentation accuracy and target completeness.

Regarding computational complexity, although PGCNet’s parameter size (25.67 MB) and GFLOPs (48.59 G) are slightly higher than some lightweight models (e.g., SegFormer, HRNet and Clusterformer), it is still much more efficient compared to large-scale models such as DeepLabv3+ (54.71 M, 243.31 G) and ConvNeXt (60.13 M, 233.5 G). Among all models, PGCNet ranks fourth in terms of parameters and computational cost, indicating that the proposed method achieves high accuracy while maintaining relatively low computational and storage requirements.

**Figure 7:**
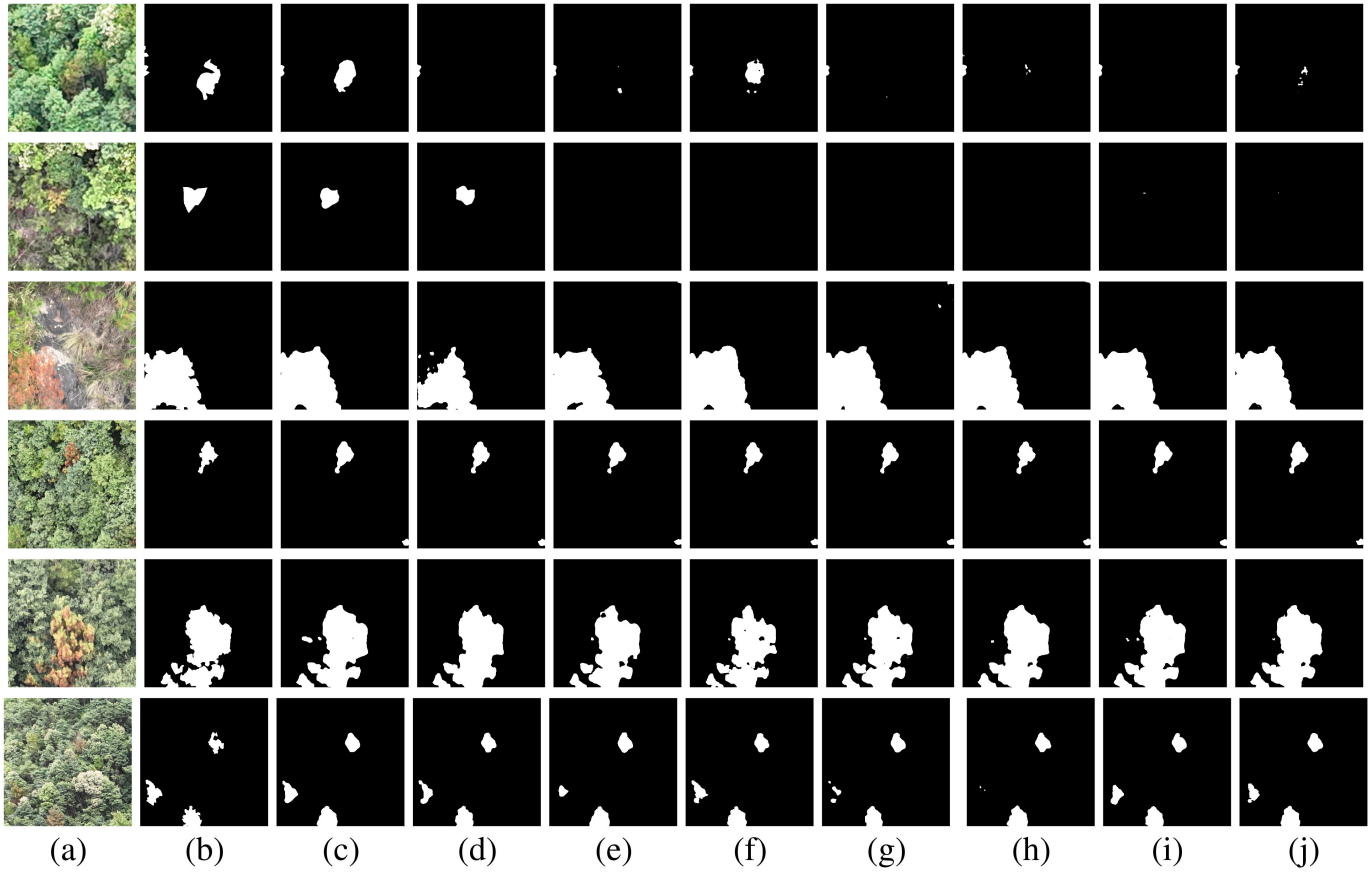
Remote sensing images, groundtruth, and segmentation maps of the proposed PGCNet and other models that achieve above 76% of IoU. **(a)** Remote sensing images. **(b)** Groundtruth. **(c)** PGCNet (79.75%). **(d)** Deeplabv3+ (77.94%). **(e)** PSANet (77.28%). **(f)** Segformer (76.27%). **(g)** ConvNext (79.33%). **(h)** HrNet (78.17%). **(i)** PSPNet (77.22%). **(j)** Clusterformer (76.29%).

2) Qualitative Evaluation: To provide a more intuitive demonstration of the effectiveness of the proposed model, the segmentation results generated by PGCNet were compared with those of other baseline models. As shown in **Figure 7**, for trees in the mid-to-late stages of PWD infection (row 4 and 5), where disease features are pronounced and less affected by lighting, all models achieve relatively good recognition. At this stage, the infected trees exhibit clear differences in features and textures compared to healthy trees, allowing most models to learn relevant features effectively.

However, for early-stage infected trees (row 1), only PGCNet and Transformer-based models (e.g., SegFormer) are capable of partial segmentation. This indicates that Transformer backbones, leveraging their long-range dependency modeling, can better extract global contextual information to identify subtle disease features, whereas CNN-based models, which rely on local feature modeling, perform weaker in this aspect.

For images with interfering deadwood and inconspicuous disease features (row 2) or where background colors are similar to disease features (row 3), some networks exhibit missed detections or misclassify background regions as diseased areas, reflecting limitations in recognizing early-stage or atypical infected trees. Additionally, for small disease targets with less pronounced features (row 6), most networks can roughly localize disease regions but show limited boundary segmentation accuracy, which may affect subsequent assessments of disease severity.

In contrast, PGCNet consistently demonstrates superior performance. Across all presented images, the segmentation maps generated by PGCNet are more precise, with fewer mislabelings or omissions compared to other models. These qualitative results are largely consistent with the quantitative evaluation in **Table 1**, further validating the effectiveness of the proposed PGCNet.

### 3.4 Ablation study

#### 3.4.1 Backbone ablation study

To evaluate the impact of different backbone networks on the segmentation performance of PWD-infected trees, this study compared several backbone architectures, including Xception Chollet (2017), ResNet50 He et al. (2016), SWin Liu et al. (2021), SWinv2 Liu et al. (2022a), and CSWin Dong et al. (2022). The experimental results are summarized in **Table 2**.

**Table 2:** Backbone ablation results.

Model	IoU(%) ↑	Precision(%) ↑	Recall(%) ↑	F1-score(%) ↑	Model size(MB) ↓	GFLOPs(G) ↓
Xception	77.94	**90.72**	84.68	87.60	54.71	243.31
ResNet50	72.95	86.83	82.03	84.36	40.34	110.37
SWin	74.70	87.58	83.56	85.52	34.83	29.67
SWinv2	74.25	87.66	82.92	85.22	28.43	**24.25**
CSWin	**78.92**	88.89	**87.55**	**88.22**	**27.24**	43.60

The bold values indicate the best results, and the underlined values represent the second-best results.

The results indicate that when CSWin Transformer is employed as the backbone network, it achieves the best performance in terms of IoU, Recall, and F1-Score, while maintaining the smallest model size, thus achieving a favorable balance between accuracy and efficiency. Although Xception attains the highest Precision, its model size is relatively large and the inference computational cost is high, which is not conducive to deployment and resource integration. For SWin/SWinv2 Transformers, the model size is relatively compact and inference speed is faster, yet their performance metrics are inferior. Traditional CNN architectures, such as ResNet50, show the lowest performance and are insufficient to meet the current high-precision segmentation requirements. Therefore, CSWin Transformer was selected as the backbone network in this study, as it achieves a desirable balance among accuracy, model size, and computational complexity.

#### 3.4.2 Synergistic effects of different combinations of the proposed modules

To further analyze the effectiveness of the proposed model’s structural design, we conducted a series of progressive ablation experiments based on the DeepLabv3+ framework, focusing on three key dimensions: feature extraction, semantic fusion, and attention mechanisms. Specifically, we sequentially introduced the CSWin Transformer backbone, the PGFM fusion module, and the CAR-ASPP context enhancement module to construct progressively improved network variants. These experiments were conducted on our self-collected dataset to evaluate both the individual contributions and the synergistic effects of each module. The comprehensive performance of each model in terms of semantic segmentation accuracy, model size, and computational complexity is summarized in **Table 3**.

**Table 3:** Ablation study results.

Model	IoU(%) ↑	Precision(%) ↑	Recall(%) ↑	F1-Score(%) ↑	Model size(MB) ↓	GFLOPs(G) ↓
DeepLabv3+(Baseline)	77.94	90.72	84.68	87.60	54.71	243.31
Baseline + CSWin (Two-branch)	78.92	88.89	87.55	88.22	27.24	43.60
Baseline + CSWin (Four-branch)	79.45	88.74	88.36	88.55	28.12	58.10
Baseline + CSWin + PGFM	79.51	87.62	89.58	88.59	27.87	49.21
Baseline + CSWin + CAR-ASPP	79.52	88.74	88.44	88.59	25.92	57.49
Baseline + CSWin + PGFM + CAR-ASPP	79.75	88.97	88.50	88.73	25.67	48.59

The baseline model used Xception as the backbone, achieving an IoU of 77.94% and an F1 score of 87.60%, with a floating-point computational cost of 243.31 GFLOPs and a model size of 54.71 MB. In the DeepLabV3+ baseline, the backbone consists of two branches to extract low-level and high-level features separately. To enhance the model’s capacity for long-range dependency modeling, we first replaced the Xception backbone with a two-branch CSWin Transformer. This structure significantly improved feature representation while maintaining efficiency, increasing IoU to 78.92% and F1 to 88.22%, with respective gains of 0.98 and 0.62 percentage points; GFLOPs decreased to 43.60 G, and the model size was reduced by half, demonstrating the benefits of the backbone replacement.

When the backbone was further extended to a four-branch configuration, model performance improved again, with IoU reaching 79.45% and F1 increasing to 88.55%, validating the effectiveness of multi-branch feature extraction for multi-scale context modeling. On this basis, the proposed Progressive Guidance Fusion Module (PGFM) was introduced. PGFM progressively guides the interaction and fusion of multi-scale semantic features, enhancing the model’s perception of pine wilt disease in complex backgrounds. After incorporating PGFM, IoU increased to 79.51%, achieving superior multi-scale feature integration while maintaining a low computational cost (49.21 GFLOPs). Compared with a direct concatenation approach in the four-branch structure, PGFM slightly improved accuracy while effectively reducing both parameter count and computational overhead.

Subsequently, to further enhance the contextual feature representation, the Context-Aware Residual ASPP (CAR-ASPP) module was introduced. This module strengthens multi-scale context modeling via an adaptive receptive field mechanism, achieving higher precision while reducing model parameters. Ultimately, when PGFM and CAR-ASPP were used jointly, the model achieved an IoU of 79.75% and F1 of 88.73%, representing improvements of 1.81 and 1.13 percentage points over the baseline, while reducing GFLOPs by approximately 80.0% and parameter count by 53.1%. The combined performance of the three modules surpassed any individual or partial combination, demonstrating their complementarity in feature representation, semantic fusion, and contextual understanding.

**Figure 8:**
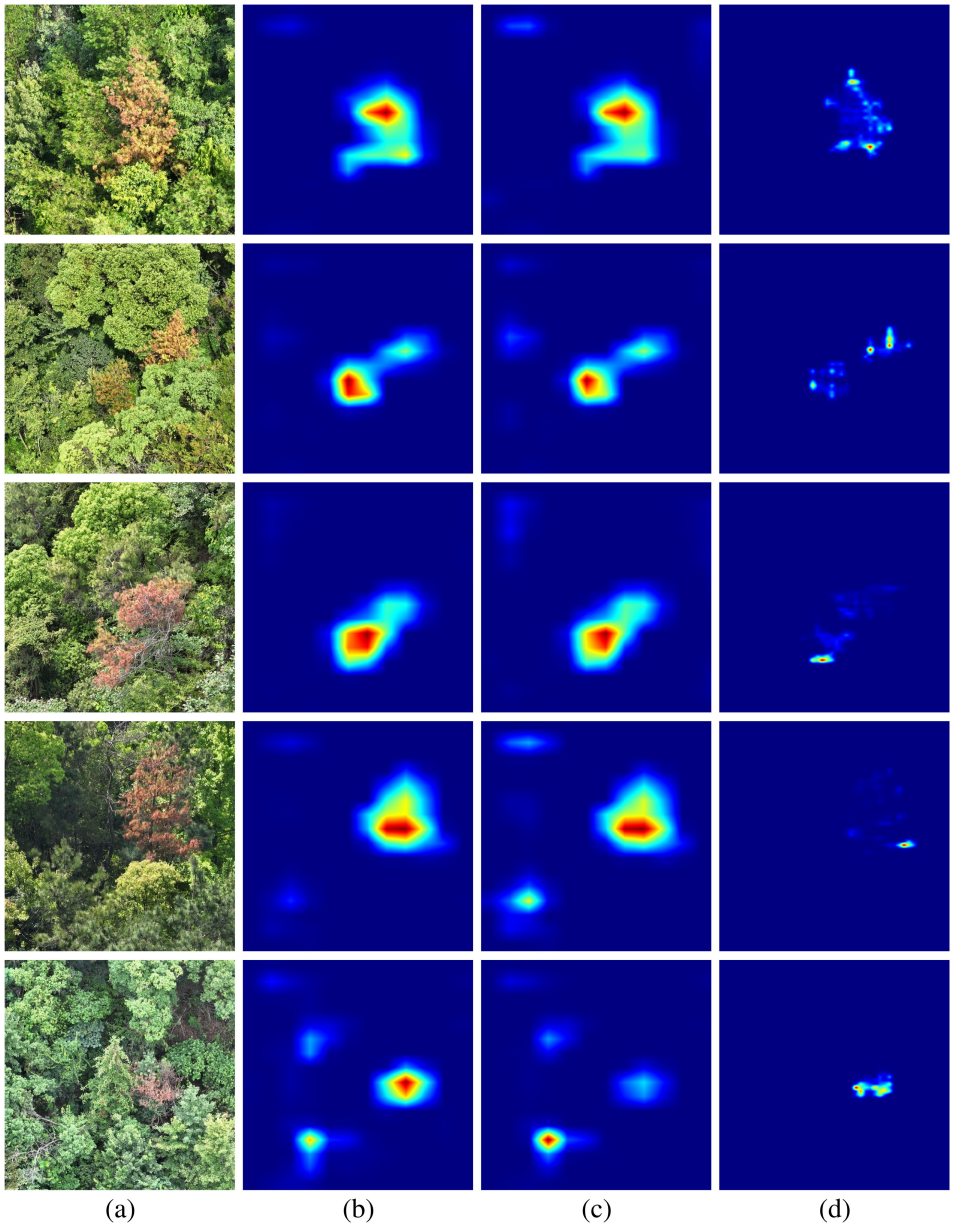
Heatmaps of model attention regions. (**(a)** original remote sensing images; **(b)** responses after integrating the CA attention mechanism into the CAR-ASPP module; **(c)** responses of the CAR-ASPP module without attention; **(d)** responses from the original baseline ASPP.).

To visually validate the effectiveness of the introduced attention mechanism, we generated heatmaps comparing the feature responses of the proposed model and the baseline during key feature extraction stages, as shown in **Figure 8**. The first column (a) presents the original remote sensing images. The second column (b) shows the feature responses of PGCNet after incorporating the Channel Attention (CA) mechanism into the high-level semantic feature extraction module CAR-ASPP. The third column (c) depicts the CAR-ASPP responses without the attention mechanism, and the fourth column (d) shows the high-level semantic feature responses extracted by the baseline ASPP module. In the heatmaps, regions closer to red indicate stronger feature responses, while blue indicates weaker responses.

From the visualization, it can be observed that adding the attention mechanism within the same network structure (b) significantly concentrates high-response regions on pine wilt disease areas, while reducing redundant responses in background regions. Compared with the version without attention (c), the model demonstrates higher spatial focus and greater accuracy in high-response regions. In contrast, the original baseline model (d) exhibits more dispersed responses, with high-response areas covering fewer disease regions and less precise boundary characterization.

These results indicate that incorporating the attention mechanism in PGCNet effectively guides the model to allocate more focus to critical regions while suppressing background interference, thereby enhancing the feature discriminability of pine wilt disease. This improvement is not only visually evident through clearer target focus in the heatmaps but also provides an intuitive explanation for the quantitative performance gains observed in **Table 3**.

### 3.5 Model generalization

To evaluate the generalization ability of the proposed model and its improvement modules, we conducted a set of ablation experiments on a publicly available plant leaf disease semantic segmentation dataset constructed on the Kaggle platform Rath (2023). This experiment used the augmented dataset, which contains a total of 2,940 images, including 2,500 images in the training set and 440 images in the validation set.

Using DeepLabV3+ as the baseline, we independently incorporated each proposed module and retrained the model on the plant leaf disease dataset for validation. The experimental setup was consistent with our primary dataset to ensure fairness, and the corresponding ablation results are summarized in **Table 4**.

**Table 4:** Ablation study on public dataset (Kaggle plant leaf disease dataset).

Model	IoU (%) ↑	Precision (%) ↑	Recall (%) ↑	F1-score (%) ↑
DeepLabv3+(Baseline)	87.97	93.44	93.76	93.60
Baseline + CSWin	88.92	93.59	94.69	94.14
Baseline + CSWin + PGFM	89.46	94.59	94.29	94.44
Baseline + CSWin + PGFM + CAR-ASPP	89.89	95.20	94.15	94.67

As shown in **Table 4**, the proposed modules also bring performance improvements on the public plant leaf dataset, with IoU increasing from 87.97% to 89.89%. This demonstrates that the effectiveness of the proposed modules is not limited to the pine wilt disease dataset, further confirming that the model and its components possess strong generalization capability.

**Figure 9:**
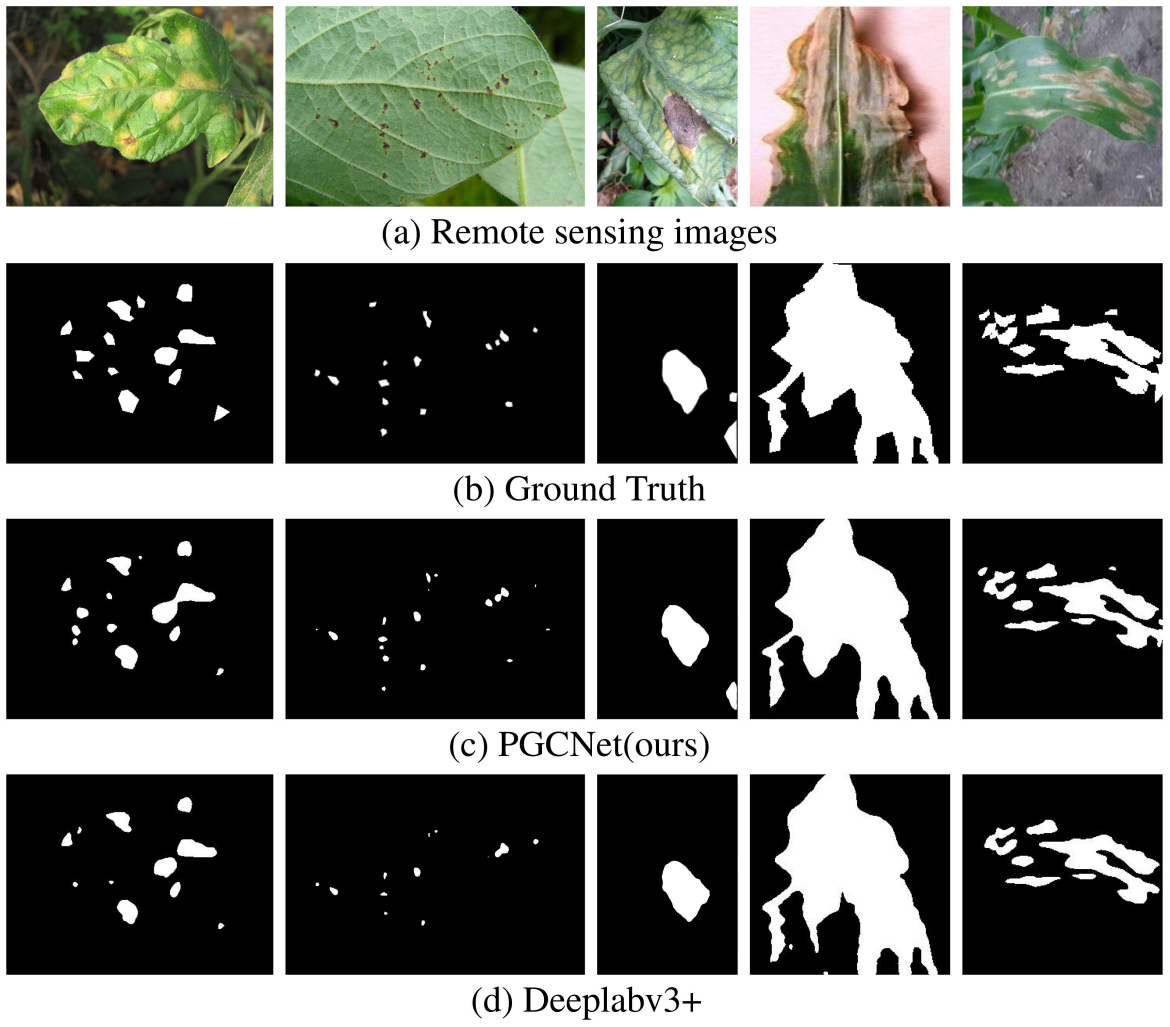
Visualization results on the public plant leaf disease dataset. **(a)** Original images. **(b)** Ground Truth masks. **(c)** Segmentation results of PGCNet. **(d)** Segmentation results of DeepLabv3+.

To visually demonstrate the effectiveness of our model on the plant leaf disease dataset, we present segmentation results from several validation samples in **Figure 9**. Even when quantitative accuracy differences are marginal, the visualization reveals that our predictions are visibly closer to the ground truth compared to the baseline. As shown in the first two rows (a) and (b), our model excels at segmenting small objects, whereas the baseline tends to miss small targets and produce blurred boundaries. This advantage stems from our integrated PGFM and CAR-ASPP modules, which enhance multi-scale feature fusion and global context capture, thereby improving accuracy for small targets and boundary regions. These results collectively confirm the model’s strong generalization capability.

## 4 Discussion

The primary objective of this study was to develop a semantic segmentation model that achieves a balanced trade-off between accuracy and computational efficiency for Pine Wilt Disease (PWD) detection. Experimental results on our self-collected dataset demonstrate that PGCNet substantially outperforms the baseline DeepLabv3+ and other mainstream architectures. Specifically, PGCNet achieves an IoU of 79.75% and an F1-score of 88.73%. Notably, these gains were attained while reducing the model size by approximately 53% (to 25.67 MB) and lowering computational complexity (GFLOPs) by 80% (to 48.59 G).

Furthermore, while PGCNet achieves a Precision of 88.97%, which is marginally lower than some general-purpose baselines, its superior Recall of 88.50% is critical for effective pest management. In the context of forest epidemic control, the implications of false negatives (missed detections) and false positives (misclassifications) are fundamentally different. A missed infected tree serves as an active transmission source, potentially leading to a resurgence of the outbreak in the subsequent season and rendering previous control efforts futile. In contrast, a false positive primarily incurs a manageable increase in verification costs. From an operational perspective, PGCNet adopts a conservative prediction strategy designed to maximize the detection of infection sources. Although this results in a certain rate of false alarms, this trade-off is operationally acceptable as it ensures that no potential hazards are overlooked prior to the deployment of field teams.

The performance advantages of PGCNet stem from the synergistic contributions of its three core components. First, the cross-shaped window attention in the CSWin Transformer enhances long-range dependency modeling, enabling more accurate discrimination of subtle lesion variations within complex forest environments. Second, the Progressive Gated Fusion Module (PGFM), combined with Spatial–Channel Synergistic Attention (SCSA), effectively bridges the representational gap between deep semantic features and shallow spatial details, significantly improving boundary reconstruction and the detection of small-scale diseased regions. The ablation experiments further confirm that PGFM contributes substantial improvements across key evaluation metrics. Third, the CAR-ASPP module employs depthwise separable atrous convolutions and a residual attention structure to achieve efficient multi-scale context extraction. This module maintains strong semantic modeling capacity while reducing parameter count and computational burden, making it well suited for real-time, lightweight UAV-based segmentation.

These architectural advantages align with recent research trends in Transformer–CNN hybrid models for PWD detection. Existing CNN-based methods—such as the work by Ding et al. Ding et al. (2025)—are often limited by restricted receptive fields, leading to difficulties in capturing fragmented or weakly textured lesion patterns. Similarly, Mask R-CNN enhanced with ConvNeXt has been shown to struggle with subtle canopy-level changes during early infection stages Wu and Jiang (2023). Pure Transformer architectures such as SegFormer Xie et al. (2021) and CSWin-based models Dong et al. (2022); Cui et al. (2025) improve long-range context modeling, yet some studies report that they may lose fine-grained local details or require higher computational costs in remote sensing applications. By reintroducing shallow structural information through PGFM, PGCNet effectively compensates for these shortcomings and achieves a more favorable balance between accuracy and efficiency compared with both purely convolutional and purely Transformer-based approaches.

Despite the encouraging results, this study is subject to limitations regarding data diversity, deployment efficiency, and sensor modality. First, the dataset was collected from a specific region in Taizhou, Zhejiang, within a limited timeframe, which constrains the full validation of the model’s generalizability across varying forest structures and seasons. Second, although PGCNet is efficient, achieving real-time inference on resource-constrained embedded UAV platforms remains a challenge. Finally, while our current RGB-based approach is robust, it misses the spectral depth offered by hyperspectral technologies, where recent advancements like Cluster Attention in SegHSI Liu et al. (2024b), Central Attention Liu et al. (2023a), and Multiarea Attention Liu et al. (2023b) have shown significant potential in optimizing computational complexity and feature extraction. Drawing on these insights, future extensions of PGCNet will aim to integrate these specialized attention mechanisms to process multispectral data, thereby enhancing detection performance in complex forest environments.

Overall, this study demonstrates that combining Transformer-based global modeling, progressive cross-layer feature fusion, and lightweight contextual modules is an effective strategy for achieving high-quality pixel-level PWD segmentation in complex forest environments. The balanced accuracy–efficiency trade-off achieved by PGCNet provides a practical technical pathway for UAV-based forest disease monitoring and offers new insights for semantic segmentation model design in natural-scene remote sensing.

## 5 Conclusion

This study proposes a segmentation model, PGCNet, specifically designed for pine wilt disease, significantly improving the efficiency and practicality of pine wilt disease detection. PGCNet adopts CSWin Transformer as the backbone for feature extraction and incorporates the CAR-ASPP module to process local information, effectively combining the advantages of CNNs and Transformers. Moreover, the study innovatively introduces the Progressive Guidance Fusion Module (PGFM) to integrate multi-scale information. Experimental results demonstrate that on the pine wilt disease detection task, PGCNet achieves an IoU of 79.75%, a Precision of 88.97%, a Recall of 88.50%, and an F1-score of 88.73%. The model also exhibits excellent lightweight characteristics, with a model size of only 25.67 MB and a computational complexity of 48.59 GFLOPs, ranking among the top-performing mainstream semantic segmentation models.

The PGCNet model effectively enhances the detection accuracy of pine wilt disease while maintaining a favorable balance between segmentation performance, model size, and inference speed, making it suitable for deployment on UAV-mounted embedded devices for efficient detection. Notably, PGCNet also demonstrates superior segmentation performance on a public plant leaf disease dataset, indicating strong generalization capability. This lays a solid foundation for extending the model to agricultural applications, providing technical support for crop disease detection, and showcasing broad application potential. Furthermore, it offers promising directions for future research.

## Data Availability

The raw data supporting the conclusions of this article will be made available by the authors, without undue reservation.
